# Farnesoid X receptor associates with β-catenin and inhibits its activity in hepatocellular carcinoma

**DOI:** 10.18632/oncotarget.2899

**Published:** 2015-02-09

**Authors:** Xijun Liu, Xingwang Zhang, Lingling Ji, Jianxin Gu, Meiling Zhou, She Chen

**Affiliations:** ^1^ Key Laboratory of Glycoconjugate Research Ministry of Public Health, Department of Biochemistry and Molecular Biology, Shanghai Medical college, Fudan University, Shanghai, China; ^2^ Department of Radiology, Zhongshan Hospital of Fudan University, and Shanghai Institute of Medical Imaging, Shanghai, China

**Keywords:** Farnesoid X receptor, β-Catenin, TCF4, Hepatocellular carcinoma

## Abstract

The association between the temporal activation of Wnt/β-catenin pathway and the spontaneous hepatocellular carcinoma (HCC) development in Farnesoid X receptor (FXR) knockout mice is not well understood. We found that Huh7 cells depleted with FXR by RNAi showed enhanced cell growth, migration and invasion *in vitro* and accelerated tumor xenografts formation in nude mice. And these phenotypes were attenuated by simultaneous knockdown of β-catenin with RNAi. Furthermore, we identified that FXR could bind with β-Catenin through AF1 domain, and disrupt the assembly of the core β-Catenin/TCF4 complex. Activation of FXR attenuated the DNA-binding activity of β-Catenin/TCF4, and subsequently, its targeting gene-*cyclin D1* expression. Importantly, FXR expression was markedly reduced in human HCC, an event which correlated with aberrant activation of β-Catenin. These data identified FXR as a negative regulator of HCC development through direct suppression of Wnt/β-catenin pathway.

## INTRODUCTION

Primary liver cancer is the fifth most common cancer in men and the seventh in women, and the third most common cause of cancer-related death worldwide. Hepatocellular carcinoma (HCC) accounts for about 85% of primary liver cancer. And its incidence increased dramatically during the last 20 years [1]. The molecular pathogenesis of HCC includes a multi-step process that results in the accumulation of various aberrant genetic and epigenetic changes. Several major oncogenic pathways involved in HCC pathogenesis, including p53/Rb, TGFβ, Met-HGF/SF and Wnt/β-Catenin signaling pathway, were identified based on the analyses of different genetic alterations [2]. Among them, aberrant activation of Wnt/β-Catenin signaling is the most frequent genetic event found in human HCC [3]. The Wnt-mediated pathway is activated through the binding of Wnt ligand to FZD receptor, an event which then triggers activation of at least three different pathways. One is the Wnt/β-catenin cascade, also called the Wnt-canonical pathway; the other two are β-catenin independent and represent examples of the non-canonical cascades including the planar cell polarity (PCP) and the Wnt/calcium pathways [4]. The canonical Wnt/FZD signaling is a critical contributor to HCC pathogenesis. 40~70% of HCC harbor nuclear accumulation of the β-catenin protein, one of the hallmarks of the Wnt/β-catenin pathway activation [5].

Farnesoid X receptor (FXR; NR1H4), a member of the nuclear receptor superfamily, is mainly expressed in liver, intestine, kidney and, to a less extent, adipose tissue [6]. FXR regulates a wide variety of genes critically involved in the control of bile acid, lipid, and glucose homeostasis [7]. Apart from its role in liver regeneration and inflammation, FXR is also involved in HCC development. FXR knockout (Fxr*^−/−^*) mice showed high incidence of spontaneous HCC development at the age of 12 to 15 months [8, 9]. Furthermore, Kim *et al*. found that, compared with age-matched WT control, Fxr*^−/−^* mice showed ~30% increase of β-catenin gene expression at 3 month, and ~100% increase at 12 month [8]. Wolfe *et al*. also reported β-catenin activation in the HCC liver from 12 to 14 month old FXR*^−/−^* mice [10]. However, the mechanisms underlying aberrant β-catenin activation in FXR deficiency mice remain unclear.

In the present study, we found that down-regulation of FXR promoted hepatocytes proliferation and carcinogenesis through β-catenin activation. FXR directly bound with β-Catenin through AF1 domain, and negatively regulated the transcriptional activity of Wnt signaling by disrupting the assembly of the core β-Catenin/TCF4 complex. Subsequently, this interaction attenuated β-catenin DNA-binding activity and reduced its targeting gene *cyclin D1* expression. Moreover, FXR expression was inversely correlated with β-Catenin activity in HCC.

## RESULTS

### Loss of FXR induced oncogenic behavior mediated through Wnt/β-catenin signaling in hepatoma carcinoma cell lines

**Figure 1:**
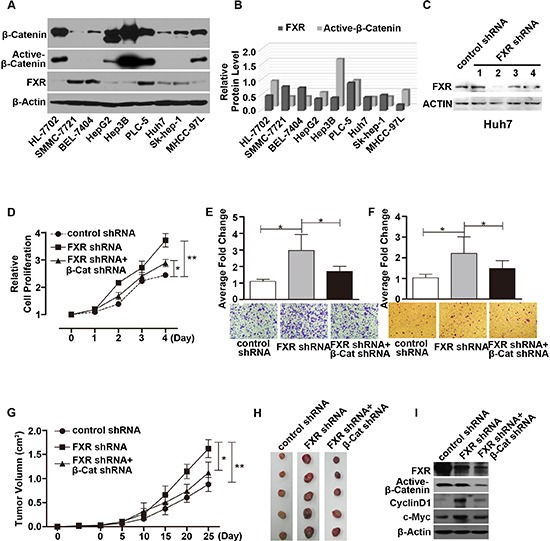
Loss of FXR induced oncogenic behavior via Wnt/β-Catenin signaling in Huh7 cells **(A)** Expression of FXR, β-Catenin and active-β-Catenin in 9 hepatoma carcinoma cell lines. Western blot of FXR, β-Catenin and active-β-Catenin was performed using total cell lysates from 9 hepatocyte cell lines. β-Actin was used as a loading control. **(B)** Quantification of Western blot analysis (normalized by β-Actin). FXR expression level was negatively correlated with active-β-Catenin level in 9 hepatocyte cell lines. **(C)** Specific knockdown of FXR was confirmed by western blot in Huh7 cells. **(D, E and F)** Silencing FXR expression by FXR shRNA #2 in Huh7 cells promoted cell growth (D), migration (E) and invasion (F) as detected by MTT, Transwell migration and Matrigel invasion assays respectively. **(G and H)** Time course of xenograft growth in nude mice. Nude mice were subcutaneously injected with Huh7 cells infected with either control shRNA, or FXR shRNA#2, or FXR shRNA#2 in combination with β-Catenin shRNA. Tumor volume was measured and tumor mass was excised and imaged at indicated time after injection. Huh7/FXR shRNA cells displayed accelerated tumor growth. And this effect was attenuated by simultaneous knocking down β-Catenin expression. **(I)** Expression of FXR, Active-β-Catenin, CylinD1 and c-Myc in the excised tumors from nude mice was determined by Western blot. Error bars represent ± SEM from three independent samples. *, *p* < 0.01; **, *p* < 0.001.

Protein expression level of FXR, and β-Catenin was determined in nine different hepatocarcinoma cell lines. As shown in Figure 1A and 1B, FXR expression level is the highest in PLC-5, the lowest in MHCC-97L and median in Huh7 cell line. Interestingly, the expression profile of active-β-Catenin negatively correlated with FXR expression in these nine cell lines.

piLenti-FXR/shRNA-GFP#2, which was more efficient in knocking down FXR expression (Figure 1C), was selected from four independent small interfering RNAs (siRNA) for the following experiments. Huh7 cell line was infected with FXR/shRNA-GFP#2 lentiviral particles to generate stable FXR knockdown cell line. As shown in Figure 1D, stable suppression of FXR significantly accelerated cell growth by about 2 times compared with the control on day 4. Down-regulation of FXR also induced significant increase in cell migration (Figure 1E) and invasion (Figure 1F). And simultaneous knockdown of β-Catenin attenuated the FXR loss of function induced cell over proliferation, migration and invasion (Figure 1D–1F). The behavior of Huh7/FXR shRNA cell line was further investigated in nude mice. Notably, FXR knockdown significantly enhanced tumorigenesis of Huh7 cells (Figure 1G). Compared with control, the size of tumor xenograft developed from Huh7/FXR shRNA cell line increased around 2 fold on day 25. And, simultaneous knockdown of β-Catenin and FXR to a large extent reversed FXR knockdown induced accelerated tumor growth (Figure 1G and 1H). Furthermore, down-regulation of FXR induced β-Catenin target genes, *Cylin D1* and *c-Myc*, overexpression. And the induced overexpression was partially reversed by down-regulation of β-Catenin by shRNA (Figure 1H).

FXR-mediated tumor inhibition was further studied with HCCLM3 cells stably over-expressing FXR. As shown in Figure S1A–S1C, overexpression of FXR dramatically inhibited cell proliferation, migration and invasion. However, these inhibitory effects were partially rescued with Wnt3a over-expression. In nude mice xenograft model, HCCLM3-FXR cells exhibited significantly reduced tumor growth. While introducing of Wnt3a markedly reversed the FXR inhibitory effect (Figure S1D and S1E). Furthermore, overexpression of FXR suppressed β-Catenin target genes, *Cylin D1* and *c-Myc*, expression. And the decreased expression was recovered by simultaneous Wnt3a overexpression (Figure S1F).

Taken together, it was demonstrated that FXR enhances the tumorigenesis of HCC. And this effect was mediated by Wnt/β-Catenin pathway.

### FXR directly interacts with β-catenin through AF1 domain

**Figure 2:**
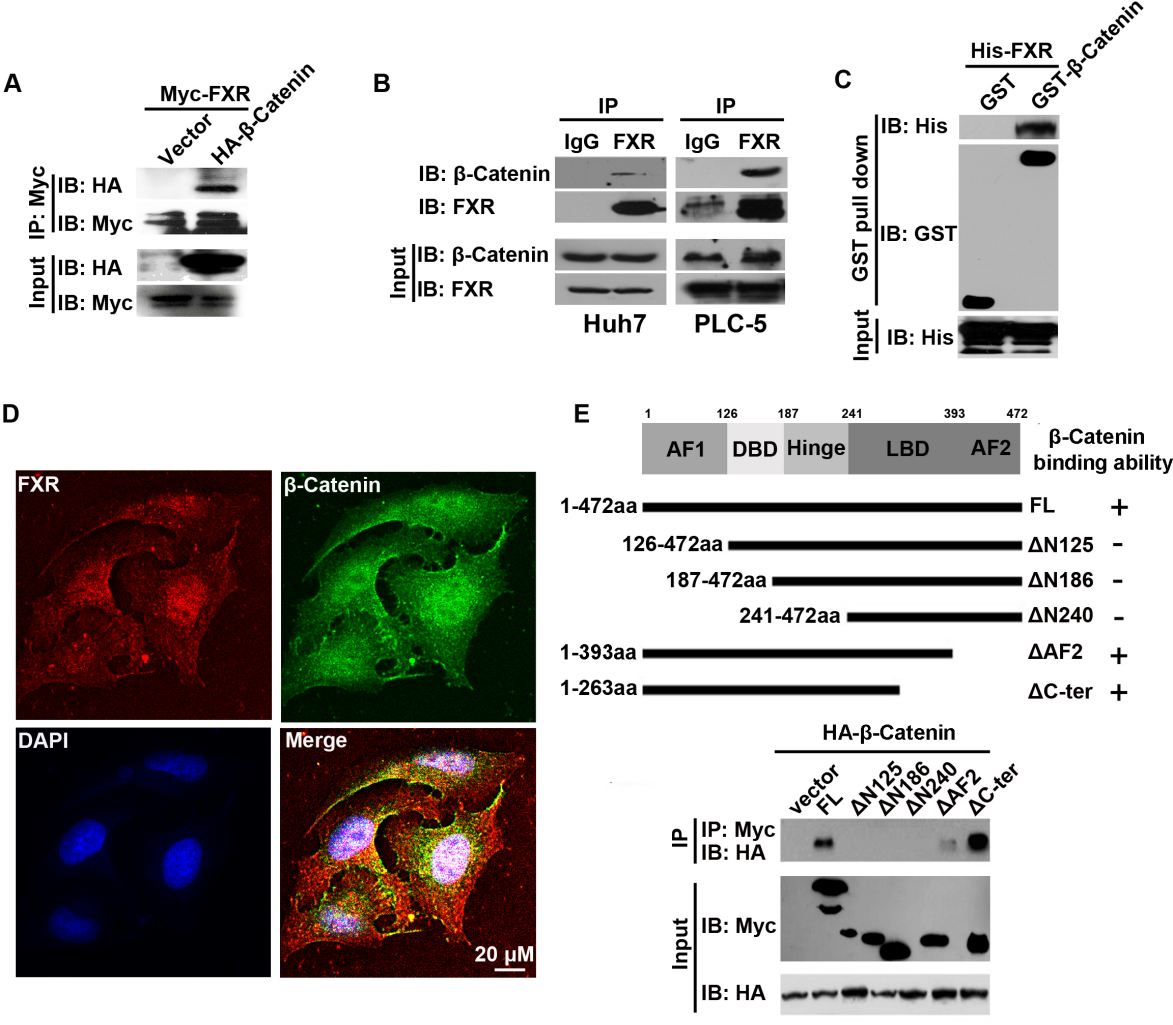
AF1 domain of FXR was required for the direct interaction between FXR and β-Catenin **(A)** Association of FXR with β-Catenin was confirmed by *in vitro* co-immunoprecipitation. Immunoprecipitation was carried out using anti-Myc agarose in lysates from HEK293T cells co-transfected with Myc-FXR and HA-β-Catenin or vector. β-Catenin was detected using antibodies against HA. **(B)** Co-immunoprecipitation of endogenous FXR and β-Catenin in Huh7 and PLC-5 cells. Immunoprecipitation was carried out in Huh7 or PLC-5 cell lysates using anti-FXR antibody, followed by IgG agarose incubation. After washing, bound β-Catenin was detected by Western blot. **(C)** GST pull-down assay. Purified His-FXR was incubated with Sepharose 4B beads pre-incubated with GST or GST-β-Catenin. After washing, bound β-Catenin was detected by Western blots. GST-β-Catenin but not GST pulled down His-FXR, indicated the direct interaction between FXR and β-Catenin. **(D)** Co-localization of FXR and β-Catenin in PLC-5 cells. PLC-5 cells were double stained with monoclonal anti-FXR antibody and polyclonal β-Catenin antibody, followed by, rhodamine-conjugated anti-mouse IgG (FXR), fluorescein-labeled anti-rabbit IgG (β-Catenin), and additional staining of nuclei with 4′,6-diamidino-2-phenylindole (DAPI). The co-localization of FXR and β-Catenin was demonstrated by the yellow color in the merged image. Scale bar = 20 μm. **(E)** FXR mutants. Diagram showed various FXR functional domains, including the AF-1 domain, the DNA binding domain (DBD), the hinge domain, the ligand binding domain (LBD), and the AF-2 domain. Solid bars indicated regions retained in the mutants. Immunoprecipitation was carried out using anti-Myc agarose from lysates of HEK293T cells co-transfected with HA-β-Catenin and respective Myc tagged FXR deletion mutants. HA-β-Catenin co-immunoprecipitated with FXR constructs that contain AF1 domain.

To identify the molecular mechanisms through which FXR regulates Wnt/β-Catenin signaling, we tested whether FXR interacts with β-Catenin. HEK293 cells were transiently co-transfected with Myc-FXR and HA-β-Catenin. The interaction between FXR and β-Catenin was confirmed by co-immunoprecipitation. We found that HA-β-Catenin could be detected in Myc immunoprecipitated cell lysate collected from Myc-FRX co-transfected cell line but not from control (Figure 2A). The interaction was further validated by co-immunoprecipitation assay with endogenous FXR and β-Catenin in both Huh7 and PLC-5 cells (Figure 2B). To test whether FXR directly binds with β-Catenin, we purified His-FXR and GST-β-Catenin and performed GST pull down assay. Purified His-FXR could bind to GST-β-Catenin but not GST protein control (Figure 2C). In addition, FXR and β-Catenin were found to co-localize in both cytoplasm and nucleus in Huh7 cells using confocal microscopy (Figure 2D).

The structural feature of FXR is characterized by N-terminal AF1 domain, a DNA binding domain (DBD), a hinge domain, a ligand binding domain (LBD) and a C-terminal AF2 domain. To identify the specific domain that mediates the interaction with β-Catenin, a series of deletion mutants, including FXR(Δ125), FXR(Δ186), FXR(Δ240), FXR(ΔAF2) and FXR(ΔC-ter), were generated (Figure 2D). Myc tagged full length FRX and its various mutants were co-transfected with HA-β-Catenin into HEK293 cells. And their interaction with β-Catenin was tested with co-immunoprecipitation assay. We found that the full length FXR and its mutants containing the N-terminal AF1 domain, including FXR(ΔAF2) and FXR(ΔC-ter), maintained the capability to bind with β-Catenin. Whereas, the other FXR mutants without N-terminal AF1 domain, including FXR(Δ125), FXR(Δ186), and FXR(Δ240), lost the β-Catenin binding ability (Figure 2E). Thus, the N-terminal AF1 domain of FXR is required for the interaction between FXR and β-Catenin.

### FXR represses β-catenin mediated transcriptional activity

**Figure 3:**
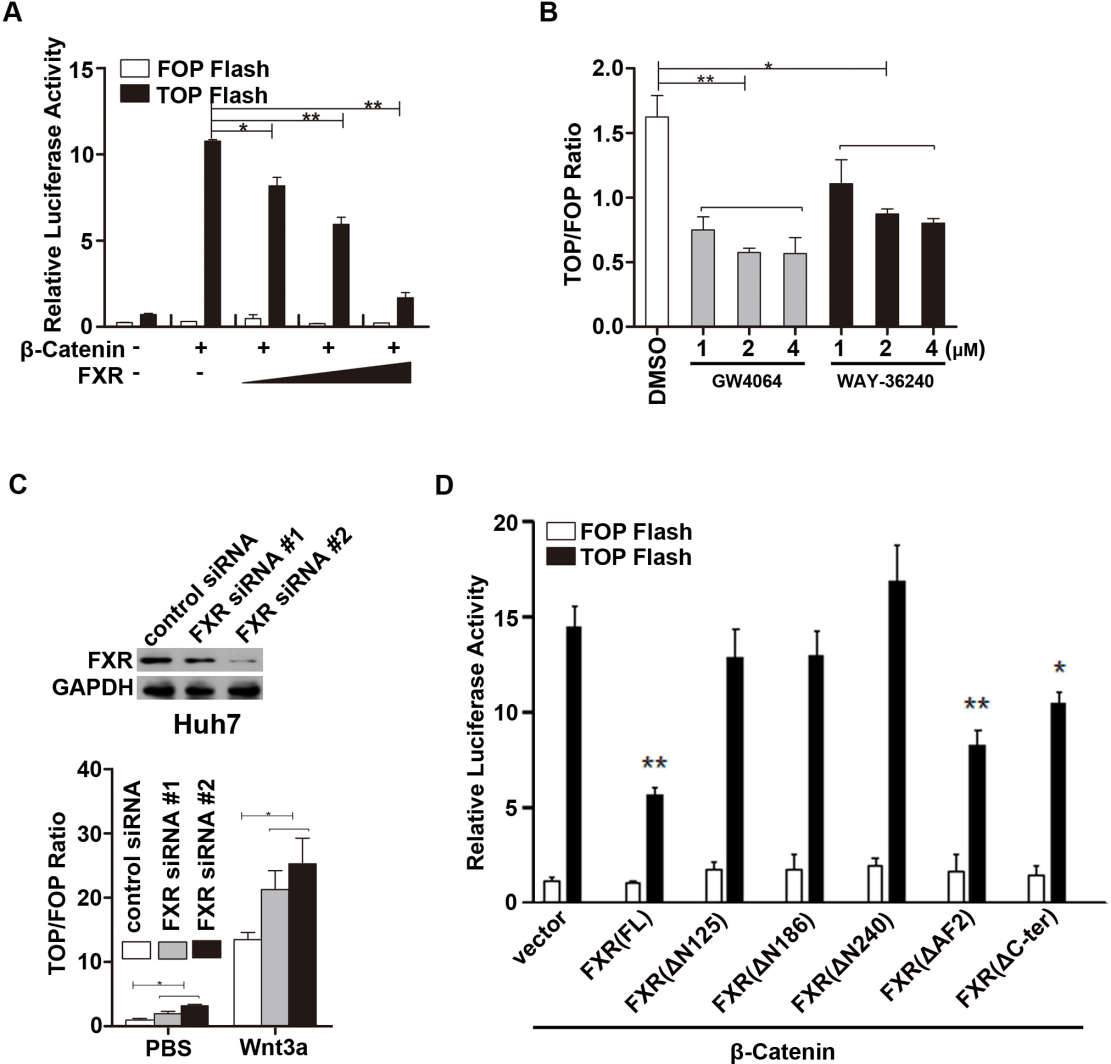
FXR repressed β-Catenin mediated transcriptional activity **(A)** FXR repressed β-Catenin mediated transcriptional activity in a dose dependent manner. HEK293T cells were transfected with TOPflash reporter (0.1 μg DNA), wild type β-Catenin and/or FXR (0.1, 0.5 and 1.0 μg DNA). Luciferase activity was measured 24 hours after transfection. FOPflash was used as negative control. **(B)** FXR agonists repressed β-Catenin mediated transcriptional activity. HEK293 cells were transfected with TOPflash reporter and pRL-TK plasmid, and then treated with FXR agonists, GW4064 or WAY-36240, ranging from 1–4 μM. Luciferase activity was measured 12–24 hours post treatment. TOPflash/FOPflash activity was normalized to that of the controls. **(C)** Down-regulation of FXR promoted β-Catenin mediated transcriptional activity. Upper panel: Specific knockdown of FXR with siRNA oligo duplexes was confirmed by western blot in Huh7 cells. FXR siRNA (#1 and #2) or control siRNA were transiently transfected into HEK293T cells in combination with TOPflash plasmid. Cells were then treated with Wnt3a (100ng/ml). And luciferase activity was measured 24 hour after treatment. **(D)** AF1 domain of FXR was required for suppression of β-Catenin mediated transcriptional activity. HEK293T cells were transfected with TOPflash reporter (0.1 μg DNA), wild type β-Catenin and various FXR deletion mutants. Luciferase activity was measured using cell lysates 24 hours after transfection. Error bars represent ± SEM of three independent samples. *, *p* < 0.01; **, *p* < 0.001.

We then tested the effect of FXR on β-Catenin mediated transcriptional activity using a cell-based TOPflash reporter assay. TOPflash reporter, pRL-TK, β-Catenin and FXR were co-transfected into HEK293 cells. As shown in Figure 3A, FXR strongly inhibited the β-Catenin mediated luciferase activity driven from the TOPflash reporter in a dose dependent manner in HEK293 cells, while showed no effect on the control FOPflash reporter plasmid. In addition, β-Catenin mediated luciferase activity was also inhibited in Huh7 cells when treated with FXR specific agonists, GW4064 and WAY-362450 (Figure 3B). In contrast, Wnt3a induced β-Catenin mediated luciferase activity was further enhanced by knocking down FXR expression via two independent FXR siRNA in Huh7 cells (Figure 3C). These results suggested that FXR is a negative regulator of Wnt/β-Catenin transcriptional activity.

We further studied whether the N-terminal AF1 domain of FXR, which is required in mediating FXR and β-Catenin interaction, is also involved in its inhibitory effect on Wnt/β-Catenin transcriptional activity. A series of FXR deletion mutants were co-transfected with β-Catenin and TOPflash reporter into HEK293 cells. We found that the full length FXR and FXR deletion mutants containing the N-terminal AF1 domain, including FXR(FL), FXR(ΔAF2) and FXR(ΔC-ter), inhibited β-Catenin mediated transcription activity. Whereas, the other FXR mutants without N-terminal AF1 domain, including FXR(Δ125), FXR(Δ186), FXR(Δ240) had no effect on β-Catenin mediated TOPflash reporter activity (Figure 3D). These results indicated that the N-terminal AF1 domain in FXR is also required for the inhibitory activity.

### FXR attenuates β-catenin/TCF4 complex mediated cyclin D1 transcription

**Figure 4:**
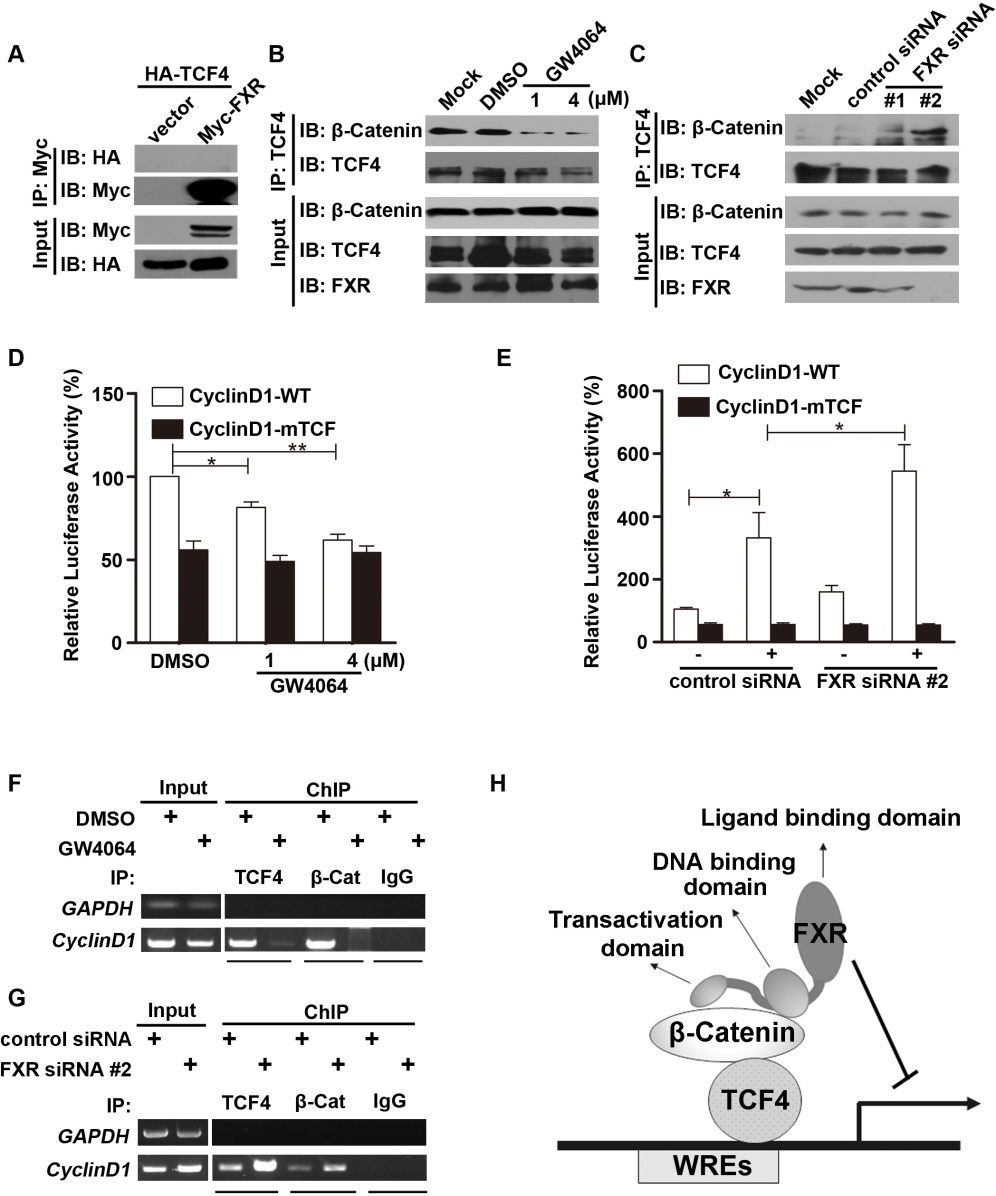
FXR attenuates β-Catenin/TCF4 complex mediated cyclin D1 transcription **(A)** FXR did not bind with TCF4. Co-immunoprecipitation was carried out with anti-Myc agarose in lysates from HEK293T cells co-transfected with Myc-FXR and HA-TCF4. TCF4 was not detected using antibodies against HA. **(B)** FXR agonist GW4064 attenuated β-Catenin/TCF4 complex formation. Co-immunoprecipitation was carried out using anti-TCF4 antibody, followed by IgG agarose incubation in lysates from Huh7 cells treated with 1 μM or 4 μM GW4064 for 24 hours. Bound β-Catenin was detected by Western blot. **(C)** Down-regulation of FXR enhanced β-Catenin/TCF4 complex formation. Immunoprecipitation was carried out using anti-TCF4 antibody, followed by IgG agarose incubation in lysates from Huh7 cells transfected with FXR siRNA (#1 or #2) or control siRNA for 24 hours. Bound β-Catenin was detected by Western blot. **(D)** FXR agonist GW4064 impaired the binding of TCF4 with *Cyclin D1* promoter. Wild type *Cyclin D1* promoter (Cyclin D1-WT; 0.1 μg), or a mutated *Cyclin D1* loss of TCF binding site (CyclinD1-mTCF; 0.1μg) and pRL-TK plasmid were transfected into HEK293 cells for 24 hours. Luciferase activity was measured using cell lysates 24 hours after cells were treated with FXR agonists GW4064 (1 or 4 μM). GW4064 impaired the binding of TCF4 with Cyclin D1-WT but not CyclinD1-mTCF. **(E)** Down-regulation of FXR promoted the binding of TCF4 with *Cyclin D1* promoter. FXR siRNA #2 or control siRNA were transfected into HEK293T cells in combination with Cyclin D1-WT or CyclinD1-mTCF. Luciferase activity was measured 24 hour after transfection. FXR siRNA #2 promoted the binding of TCF4 with Cyclin D1-WT but not CyclinD1-mTCF. **(F)** FXR agonist GW4064 reduced the association between β-Catenin/TCF4 complex and the TCF binding sites from *Cyclin D1* promoter. ChIP analysis was carried out using antibodies against TCF4, β-Catenin, or normal IgG in lysates from Huh7 cells. DNA fragment precipitates were amplified by PCR (35 or 38 cycles). **(G)** Down-regulation of FXR promoted the association between β-Catenin/TCF4 complex and the TCF binding sites from *Cyclin D1* promoter. ChIP analysis was carried out using antibodies against TCF4 or β-Catenin, or normal IgG in lysates of Huh7 cells transfected with FXR siRNA #2 or control siRNA. DNA fragment precipitates were amplified by PCR (35 or 38 cycles). **(H)** Model of mechanism for the FXR mediated repression of the canonical Wnt/β-Catenin signaling. Upon activation, FXR, through its interaction with β-Catenin, prevents β-Catenin/TCF4 complex formation, subsequently represses transcription of its downstream target genes, such as cyclin D1. Error bars represent ± SEM of three independent samples. *, *p* < 0.01; **, *p* < 0.001.

Next, we investigated whether FXR also interacts with the other core transcription factor, TCF4. HEK293 cells were transiently transfected with Myc-tagged FXR and HA-tagged TCF4. The interaction between FXR and TCF4 was tested with co-immunoprecipitation assay. We found that HA-TCF4 was not presented in the cell lysate immunoprecipitated with Myc agarose (Figure 4A). β-Catenin and TCF4 protein level were unaffected by FXR specific agonist GW4064 in Huh7 cells (Figure 4B, input). We then investigated whether FXR regulates the stability of β-Catenin-TCF4 complex. We found that, upon FXR activation in GW4064 treated Huh7 cells, the binding between β-Catenin and TCF4 was decreased (Figure 4B). In contrast, when FXR expression was depleted by two independent siRNA in Huh7 cells, the interaction between β-Catenin and TCF4 was enhanced (Figure 4C).

Furthermore, whether FXR affects specific β-Catenin-TCF4 complex targeting *Cyclin D1* gene transcriptional activity was examined using a TOPflash reporter assay. Wild type *Cyclin D1* promoter (Cyclin D1-WT; 0.1 μg), or a mutated *Cyclin D1* loss of TCF binding site (CyclinD1-mTCF; 0.1 μg) and pRL-TK plasmid were transfected into Huh7 cells, upon FXR activation by GW4064, relative Cyclin D1-WT promoter activation was reduced, while the CyclinD1-mTCF promoter activation was not altered (Figure 4D). In contrast, when FXR expression was depleted by its siRNA in Huh7 cells, the relative Cyclin D1-WT, but not CyclinD1-mTCF, promoter activation increased (Figure 4E).

Next, ChIP assays were performed to test how FXR regulates the interactions between TCF4/β-Catenin complex and *Cyclin D1* promoter. Binding of both TCF4 and β-Catenin to the *Cyclin D1* promoter was attenuated after FXR specific agonist GW4064 treatment in Huh7 cells (Figure 4F). While formation of the β-Catenin-TCF4 complex on the *Cyclin D1* promoter was increased in Huh7 cells expressing FXR siRNA#2 (Figure 4G).

These results indicated that FXR repressed Wnt/β-Catenin transcriptional activity by direct interaction with β-Catenin through AF1 domain. And this interaction attenuated the β-Catenin-TCF4 complex formation, and subsequently, the association of β-Catenin-TCF4 with Wnt response elements and the corresponding transcriptional activity (Figure 4H).

### FXR expression is frequently reduced in human hepatocarcinoma, correlating with elevated β-catenin activation

**Figure 5:**
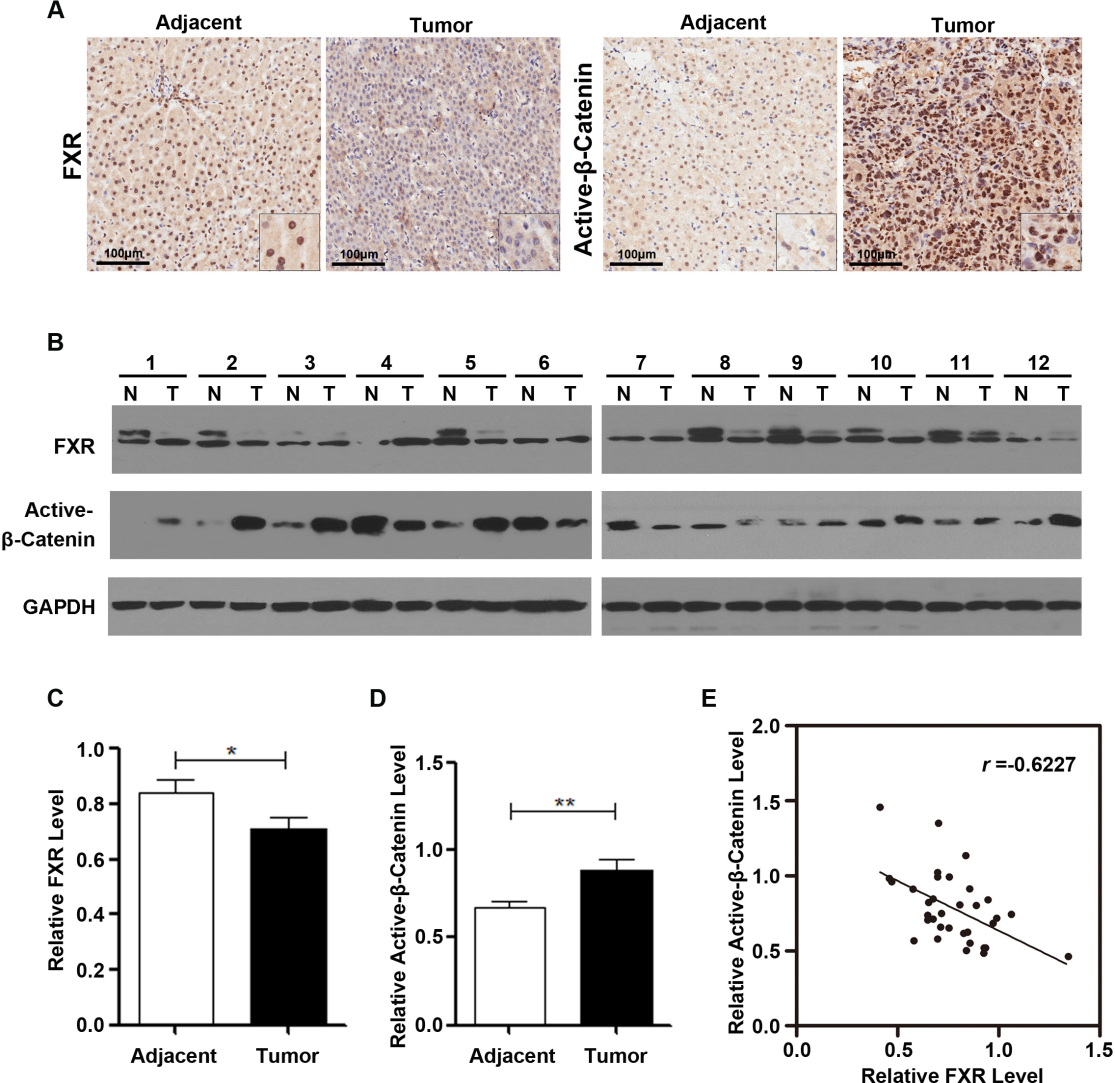
FXR expression was frequently reduced in human Hepatocarcinoma, correlating with elevated β-Catenin activation **(A)** Immunohistochemical staining of FXR and active-β-Catenin in the sections of a representative HCC sample. *Inset*, 10X of the same section. FXR expression was identified mainly in the nucleus of the adjacent tissue and negative in the tumor. In contrast, active-β-Catenin staining was negative in the adjacent tissue, whereas, strongly positive in tumor nucleus. **(B)** Western blot analysis of FXR and active-β-Catenin in 12 paired representative HCC tissues (T) and non-tumor tissues (N) **(C)** Quantification of FXR protein level in HCC. **(D)** Quantification of active-β-Catenin protein level in HCC. **(E)** The correlation between FXR and active-β-Catenin protein level in paired HCC and non-tumor tissues. Pearson's correlation coefficients: *p* = 0.0092, *r* = −0.6227. Data was presented as mean ± SEM of 3 independent experiments.*, *p* < 0.01, **, *p* < 0.001.

To address whether FXR and β-Catenin activation are involved in human hepatocarcinoma development, we performed immunohistochemistry staining in 8 paired formalin-fixed paraffin-embedded human hepatocarcinoma tissue samples. As shown in Figure 5A, the expression of FXR was significantly reduced in tumor sample compared with adjacent tissue. And it was also observed that FXR was mainly found in the nuclei of the adjacent tissue. In contrast, overexpression of active-β-Catenin was detected in in tumor compared with adjacent tissue. And it was located in both nucleus and cytoplasm.

To further understand the correlation between the expression of FXR and active-β-Catenin, we investigated the expression level of FXR and active-β-Catenin by Western blot in HCC (T) and matched adjacent tissues (N). Out of 12 paired samples, 8 pairs showed decreased FXR expression and increased active-β-Catenin expression in tumor tissue when compared with matched adjacent tissue (Figure 5B–5D). Association analysis of 34 HCC tissues indicated that the expression of FXR and active-β-Catenin was negatively correlated (Pearson's correlation coefficients *r* = −0.6227; Figure 5E).

Taken together, these data provided the evidence that, in human hepatocarcinoma, FXR expression is frequently reduced, along with elevated β-Catenin activation.

## DISCUSSION

In this study, we identified a crosstalk between FXR and canonical Wnt signaling pathway. The expression of FXR was significantly downregulated and was inversely correlated with β-Catenin activation in HCC. FXR directly bound with β-Catenin and inhibited the formation of β-Catenin/TCF4 complex, and subsequent transcriptional activity. Our data demonstrated a novel mechanism through which FXR exhibited tumor suppressive activity.

We showed that FXR expression level was significantly downregulated in human HCC, and that loss of FXR promoted tumorigenic behaviors in hepatoma carcinoma cell line Huh7; whereas, stably over-expressing FXR inhibits tumor growth in HCCLM3 cells. Consistent with our observation, previous works also discovered reduced FXR expression in cholangiocarcinoma, biliary tract carcinoma and tumor sample from HCC patients [11–14]. In addition, a recent report showed a tumor-stage dependent reduction of FXR at both mRNA and protein level in human colon carcinoma [15, 16]. It seems that FXR deregulation is a common mechanism associated with tumorigenesis of several tissue types. On the other hand, FXR^−/−^ mice were more prone to develop hepatocellular carcinoma, along with elevated β-Catenin expression. We found that FXR negatively correlated with β-Catenin activation in both hepatoma carcinoma cell lines and human hepatocarcinoma tissue. And reduced FXR promoted tumorigenic behaviors was partially reversed by simultaneous knockdown of β-catenin. These data suggests that β-Catenin is a critical component in this process. Oncogenic Wnt/β-Catenin signaling is likely to be one of the important factors that contributes to the development and maintenance of HCC during loss of FXR function.

Wnt/β-Catenin signaling is highly conserved from nematodes to humans, and is essential in various normal cellular processes such as development, growth, survival, regeneration and self-renewal [17]. Mutations in Axin, APC and β-Catenin were involved in dysregulation of Wnt signaling [18, 19]. Aberrantly activated Wnt/β-catenin pathway was frequently found in HCC [20]. And there is a linkage between upregulation of β-Catenin target genes and increased transformation from hepatocyte to HCC [18, 21, 22]. Previous studies indicated that abnormal cytoplasmic and nuclear accumulation of β-Catenin exists in 20% to 40% of HCC [5]. Our results confirmed the over expression of activated β-Catenin in both cytoplasmic and nuclear from HCC tissue. And β-Catenin expression pattern is conversely correlated with FXR expression.

The crosstalk between Wnt/β-catenin ligands and members of the nuclear receptor (NR) family has been considered as a clinically and developmentally important research area of cancer biology [23]. For example, activated β-Catenin has been shown to co-activate Retinoic Acid Receptor (RAR) associated with RAR response elements in MCF7 breast cancer cells [24]. And activation of Vitamin D receptor (VDR) could repress Wnt/β-Catenin signaling and decrease 1α, 25(OH)_2_ vitamin D3 induced tumorigenesis [25]. In the case of FXR interaction with β-Catenin, FXR appears to regulate β-Catenin activity but not affecting β-Catenin localization and protein stability since the knockdown of FXR does not affect the translocation of β-Catenin (Figure S2), and the total protein level of β-Catenin expression was not altered by either FXR activation by GW4064 or FXR depletion by FXR-siRNA in Huh7 cells. Activated β-Catenin forms a complex together with TCF4, and is recruited to the corresponding promoter region of Wnt target genes to elicit transcriptional activity [26]. We identified that FXR, a member of nuclear receptor (NR) family, directly interacted with β-Catenin *in vitro* and *in vivo*, to disrupt the formation of β-Catenin/TCF4 complex and subsequently the binding ability with corresponding promoter. Interestingly, FXR can bind with and inhibit the corresponding transcriptional activity of both wild type β-Catenin and two oncogenic β-Catenin hot spot mutants (S33A/S37A/T41A/S45A and ΔN90) (Figure S3). These findings suggested that β-Catenin is a pathway through which FXR affects tumorigenesis.

Among 19 Wnt ligands, Wnt3a is classified as the canonical element, leading to canonical Wnt/FZD signaling pathway activation [27]. Our study indicated that the tumorigenic behavior of HCCLM3 cells was significantly inhibited by the overexpression of FXR. While ectopic Wnt3a expression partially rescued FXR induced inhibitory effects. Activation of the canonical Wnt/FZD signaling leads to the accumulation of β-catenin in the cytoplasm and then translocation into the nucleus, where it binds to TCF/LEF transcription factors and then forms a transcriptionally active complex with CBP, Bcl9, and Pygo proteins, to promote the expression of target genes. However, Wnt3a can only partially rescue the FXR induced inhibition when the increased β-catenin cannot fully compensate the loss of free β-catenin due to FXR association. Also, it was clear from previous studies that β-Catenin is not the only pathway through which FXR affects tumorigenesis. Loss of FXR also resulted in downregulation of SHP and NDRG2. And SHP^−/−^ mice developed HCC in a time-dependent fashion [28, 29]. FXR has also been shown to block HER2/MAPK signaling, which led to reversed anti-estrogen resistance in human breast cancer cells [30]. Another study revealed that a membrane-anchored inhibitor of matrix metalloproteinase RECK is also a target gene of FXR in mouse liver [31]. Moreover, FXR also controls many hepatoprotective genes, including genes involved in detoxification of reactive oxygen species [32]. Up-regulation of β-Catenin is just one of many factors that contribute to the development of HCC upon loss of FXR function. These data suggested that FXR inhibits tumorigenesis at least partially through Wnt/β-Catenin signaling.

Our data provided a novel mechanism for FXR-mediated inhibition of tumor progression. Through competing with other co-activators for binding to β-Catenin, FXR suppressed the β-Catenin-mediated activation of the Wnt target genes, which are involved in tumor development. FXR, a new modulator of the Wnt signaling pathway, is a potential candidate as a molecular target of the Wnt signaling cascade to achieve anti-tumor effects.

## MATERIALS AND METHODS

### Clinical samples and human cell lines

HCC tissues and the paired adjacent non-tumorous tissues were obtained from Zhongshan Hospital of Fudan University. Ethical approval for human subjects was obtained from the Research Ethics Committee of Zhongshan Hospital. And informed consent was obtained from each patient. For immunohistochemistry analysis of FXR and β-Catenin expression in human liver cancer tissue specimens, 8 paired fresh frozen liver tissues and matched normal tissues were used. 19 paired fresh resections were collected for western blot analysis.

The SMMC-7721, HL-7702, BEL-7404, HepG2, Hep3B, Huh7, Sk-hep-1, MHCC-97L and HCCLM3 cell lines were obtained from the Type Culture Collection of Chinese Academy of Sciences (Shanghai, China), and were maintained in Dulbecco's modified Eagle's medium (Gibco) supplemented with 10% fetal bovine serum (Gibco) at 37°C in a humidified incubator containing 5% CO_2_. FXR agonists (GW4064 and WAY-362450 both were purchased from Selleck Chemicals, Houston TX, USA) treatment was carried out in charcoal stripped fetal bovine serum (Gibco).

### Plasmids and luciferase reporter assay

pLKO.1-shβ-Catenin and shNC (negative control), pT3-ΔN90-β-Catenin, pcDNA3-β-Catenin-MutS33A/S37A/T41A/S45A, pGL3-Cyclin D1 WT, pGL3-Cyclin D1 mTCF, SuperTOPflash/FOPflash-TCF4 luciferase reporter (under the control of eight TCF4 consensus sites) were purchased from Addgene. pReceiver-Lv105-Wnt3a was purchased from GeneCopoeia. Human FXR (NM_005123) coding region was amplified and cloned into recombinant lentivirus vector pLKO.1. The deletion mutants of Myc tagged FXR 126–472, 187–472, 241–472, 1–393, or 1–263 were amplified by PCR using primers corresponding to each mutant. The PCR products were digested by *Xho1* and *BamH1*, and then ligated into pcDNA3-Myc vector. All constructs were verified by sequencing.

We employed different approaches for transient or stable knocking down gene expression with small interfering RNA: 1) for transient suppression, synthesized siRNA oligo duplexes were transiently introduced into the cells using Lipofectamine 2000; 2) for stable suppression, shRNA was introduced into lentiviral vectors to produce lentiviral particles in HEK293T cells, and then infected Huh7 cells to generate stable gene silencing. Four independent piLenti-FXR shRNA-GFP were purchased from abm Inc (Cat. i015336). Among them, piLenti-FXR/shRNA-GFP#2, which was more efficient in knocking down FXR expression, was selected from establishing stable cell lines. Specific siRNA oligo duplexes targeting FXR (FXR siRNA#1:5′-GCGGTTGAAGCTATGTTCCTTCGTT-3′;FXR siRNA# 2:5′-GGCTCCAGGGAATCCTGCATTCTAA-3′) and the negative control siRNA were synthesized by GenePharma (Shanghai, China), and transiently introduced into the cells using Lipofectamine 2000. pLKO.1 shRNA FXR construct was derived from FXR siRNA#2 sequence and used to generate the Huh7 stable cells expressing FXR silencing in Figure S2.

Luciferase assay was performed using a Dual-Luciferase® Reporter Assay System (Promega). Briefly, 200 ng of TOPflash or *Cylin D1* promoter reporter and 10 ng of pRL-TK with or without 100nM FXR siRNA were co-transfected into Huh7 cells using Lipofectamine 2000 kit (Invitrogen, Carlsbad, CA) according to the manufacturer's instructions. 48 h after transfection, luciferase activity was measured from harvested cell lysate using luminometer. And the result was normalized by Renilla activity.

### GST pull-down assay

His-FXR (Abcam, Cambridge, MA) was purified according to the manufacturer's instructions (GE Healthcare), and then incubated with glutathione-Sepharose 4B beads bound GST or GST-β-Catenin in binding buffer (20 mM Tris-HCl (pH 7.5), 150 mM NaCl, 0.2% Nonidet P40 and 10% glycerol). After incubation at 4°C overnight, the beads were washed 6 times for 10 min in binding buffer. And proteins bound to the beads were eluted into sample buffer, followed by SDS-PAGE, and Western blots.

### Immunochemical (IHC) staining

The 6 μM thick paraffin-embedded tissues were deparaffinized and rehydrated. Antigen retrieval was performed in a steamer for 15 minutes. The section was stained with a rabbit monoclonal anti-active-β-Catenin (1:100, Cell Signaling) and a mouse monoclonal anti-FXR (1:100, Abmart, China) respectively overnight at 4°C. After washing with PBST, biotinylated secondary antibodies (1:50, Dako, Carpinteria, CA) was incubated for 2 hours at room temperature. Reactions were developed using a peroxidase-based streptavidin detection method and 3, 3′-diaminobenzidine tetrahydrochloride (DAB) (Dako, Carpinteria, CA) as chromogenic substrate. The tissue sections were mounted and examined under the CKX31 microscope (Olympus, Osaka, Japan).

### Chromatin immunoprecipitation assay

Chromatin immunoprecipitation analysis was performed using EZ ChIP kit (Millipore) according to the manufacturer's instructions. The sheared material was used for isolating input DNA. Aliquots of sheared chromatin corresponding to 1 × 10^6^ cells was incubated with anti-TCF4, anti-β-Catenin antibody (Cell Signaling), or IgG, in 300 μl ChIP buffer. DNA fragments were purified from immunopriciptates. PCR was performed with forward CCGGGCTTTGATCTTTGCTTA and reverse TCTGCTGCTCGCTGCTACTG primers, for 35 cycles (15 sec 95°C and 60 sec 60°C) to amplify a 120bp DNA fragment harboring the WRE sequence in the *Cyclin D1* promoter.

### Western blots and immunoprecipitation assays

Western blot were performed according to standard procedures. Briefly, the liver tissues or cultured cells were lysed in RIPA buffer (50 mM Tris/Cl pH 8.0, 140 mM NaCl, 1.5 mM MgCl_2_, 0.5% (v/v) Nonidet P-40). Equal amount of protein (10 ~ 80 μg) was electrophoresed by SDS-PAGE and then transferred to nitrocellulose membranes. After blocking with PBS containing 5% nonfat milk and 0.1% Tween 20 for 2 hours at room temperature, the membrane was incubated with primary antibody at 4°C overnight. After washing with PBS containing 0.1% Tween-20 3 times, 5 minutes each, the membrane was then incubated with horseradish peroxidase labeled secondary antibody for another 2 hours at room temperature. The membrane was then developed using Pierce ECL Western Blotting Substrate (Piece, Rockford, USA) and exposing to KODAK X-OMAT BT Film.

For co-immunoprecipitation, cells were lysed in lysis buffer (50 mM HEPES, pH 7.5, 150 mM NaCl, 1 mM EGTA, 10% glycerol, 1.5 mM magnesium chloride, 1% Triton X-100, 1 mM phenylmethylsulfonyl fluoride, 1 μg/ml leupeptin, and 50 units/ml aprotinin). After centrifugation at 16,000 g for 15 minutes, the lysates were immunoprecipitated with primary antibodies followed by protein A/G-Sepharose (Santa Cruz) or tag conjugated beads. After washing 5 times with lysis buffer, proteins were resolved by SDS-PAGE gels and transferred to nitrocellulose membranes for Western blot.

The primary antibodies used were purchased from Cell Signaling Technology, CA (anti-β-actin, anti-Cyclin D1, anti-c-Myc, anti-Active-β-Catenin and anti-β-Catenin antibodies, 1:1000), Santa Cruz Biotechnology, CA (anti-Myc, anti-FLAG, anti-GST, and anti-TCF4 antibodies, 1:1000), Roche Applied Science Indianapolis, IN, (anti-HA antibody, 1:5000), and Abmart, China (Anti-FXR antibody, 1:1000) respectively.

### Immunofluorescence staining and confocal microscopy

For immunofluorescence analysis, cells were fixed with 4% paraformaldehyde in PBS at 4°C for 30 minutes, washed 3 times with PBS, and permeabilized by incubation in PBS with 0.1% Triton X-100 for 10 minutes. After blocking in PBS with 10% goat serum and 0.1% Triton X-100 for 1 hour at room temperature, cells were incubated with the primary antibodies (anti-FXR, Abmart, China) monoclonal mouse antibody and anti-β-Catenin rabbit antibody (Cell Signaling) at 4°C overnight, followed by incubation with the secondary antibodies (Alexa488-conjugated anti-rabbit IgG (Invitrogen, Carlsbad, CA) and Alexa 594-conjugated anti-mouse IgG (Invitrogen, Carlsbad, CA) at room temperature for 2 hours. Coverslips were mounted onto glass slides with anti-fade mounting medium with DAPI (Vector Laboratories) and visualized with an Olympus FluoView 500 IX71 confocal microscope.

### Cell proliferation assay

Cells (5 × 10^3^ cells) were seeded in triplicate in 96-well plates and incubated for 4 days. At indicated time point, 20 μl of MTT (3-(4, 5-dimethylthiazol-2-yl)-2, 5-diphenyltetrazolium bromide (Sigma, St. Louis, MO)) solution (5 mg/ml) was added to each well. The plates were incubated at 37°C for 5 hours, followed by removing the medium, and then adding 150 μl DMSO to each well to dissolve crystals. The plates were then measured at 590 nm with a reference filter of 620 nm on a multi-well plate reader. Absorbance readings reflect the number of viable cells. To calculate relative cell proliferation, the following equation was used: relative cell proliferation= (Experiment group OD_day(n)_-Blank OD_day(n)_)/(Experiment group OD_day 0_-Blank OD_day 0_). *n* = 0, 1, 2, 3, 4 respectively; experiment group includes, (1) cells transfected with control shRNA, (2) cells transfected with FXR shRNA, and (3) cells transfected with FXR shRNA +β-catenin shRNA; blank is cell culture medium only. For all the experiment group, including cells transfected with control shRNA, the relative value is 1 only on Day 0. While on Day 1 to 4, the value for experiment group, including cells transfected with control shRNA, reflects relative proliferation compared with Day 0.

### Migration assay and invasion assay

To assess cell migration, Huh7 or HCCLM3 cells (5 × 10^5^ cells/well) were seeded on top of the 8.0 μm pore size transwell insert containing 600 μl DMEM medium in the lower chamber. Cells migrated to the other slide of membrane were stained with crystal violet and counted after 24 hours incubation.

To assess cell invasion, cells (1 × 10^5^) were plated in 8.0 μm pore size Matrigel coated transwell insert. Culture medium was changed twice a day for 3 days. After 48 hours, cells that had migrated through the Matrigel to the filter were stained with crystal violet and counted.

### Mouse xenograft model

All animal procedures were performed in accordance with protocols approved by the Animal Use Committees at Fudan University. Huh7 or HCCLM3 cells were maintained in in Dulbecco's modified Eagle's medium (Gibco) supplemented with 10% fetal bovine serum (Gibco). Cells were infected with concentrated lentivirus (multiplicity of infection = 25). Stably transfected cell clones were selected. Cells were grown at 37°C in the tissue culture incubator for two days and dissociated into single cells using dispense. Following established procedures, 2.5 × 10^6^ cells were re-suspended in 0.1 ml PBS containing 10% matrigel and then injected subcutaneously into the right dorsal flank of 4-week old nude mouse, which were obtained from the Experimental Animal Center of Fudan University. Tumors were carefully excised, measured, imaged by digital camera and collected for Western blot. Tumor volume is calculated as V (mm^3^) =(ab^2^)/2, where “a” indicates the length and “b” indicates the width of the tumor.

### Statistical analysis

Pearson's correlation coefficients (*r*) analysis was used to determine the correlation between FXR and active-β-Catenin protein level in paired HCC tissues. Data were expressed as means ± SEM. Statistical analysis was performed either by Student's t test for unpaired data or one-way ANOVA for three groups or more. *, *p* < 0.05; and **, *p* < 0.001 was considered as significant.

## SUPPLEMENTARY FIGURES


