# Supplementary material for: Farnesoid X receptor associates with β-catenin and inhibits its activity in hepatocellular carcinoma
Source: Oncotarget. 2015 Feb 9;6(6):4226–38. doi: 10.18632/oncotarget.2899 (PMC4414185; doi:10.18632/oncotarget.2899)
Supplement: Supplementary file 1 [file oncotarget-06-4226-s001.pdf]

SUPPLEMENTARY FIGURES

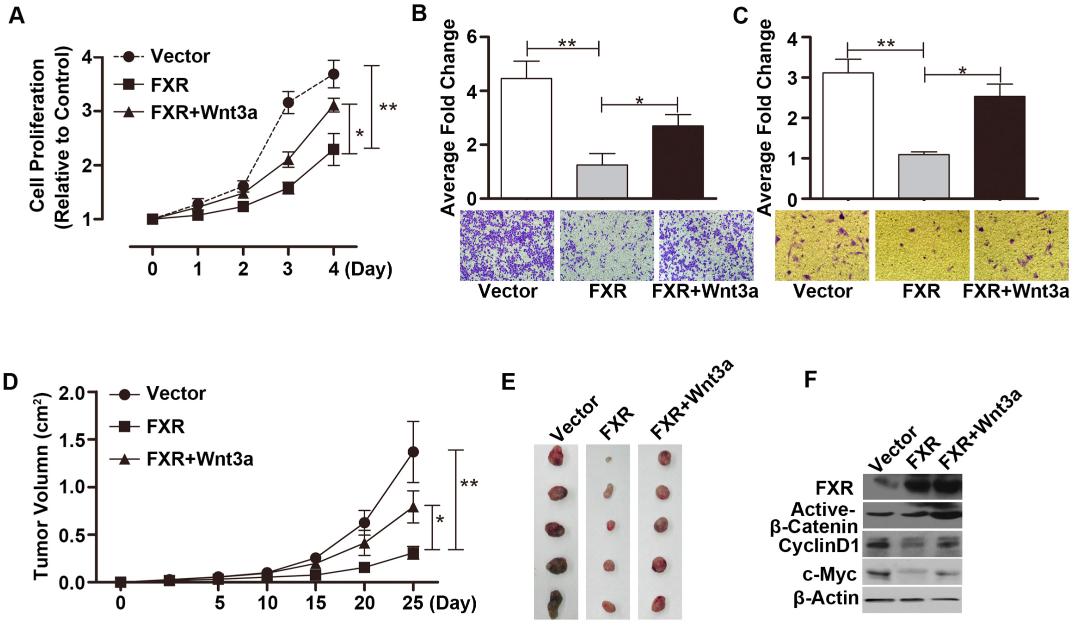

Supplementary Figure S1: Overexpression of FXR inhibited HCCLM3 cells oncogenic behavior via Wnt/β-Catenin. (A, B and C) Over-expression of FXR in HCCLM3 cells suppressed cell growth (A), migration (B) and invasion (C) as detected with MTT, Transwell migration and Matrigel invasion assay respectively. And simultaneous treatment with Wnt3a could partially reverse this effect. **(D and E)** Nude mice were subcutaneously injected with HCCLM3 cells (2.5×10^6) over-expressing either FXR, or FXR in combination with Wnt3a. Tumor volume was measured and tumor mass was excised and imaged at indicated time after injection. Ectopic FXR expression in HCCLM3 cells decreased tumor xenograft growth *in vivo*. Whereas, activation of β-Catenin by constitutively expressing Wnt3a reversed FXR mediated tumor suppression. **(F)** Expression level of FXR, active-β-Catenin, CylinD1 and c-Myc in the excised tumor samples from nude mice was determined by Western blot. Error bars represent ± SEM from three independent samples. *, $p < 0.01$; **, $p < 0.001$.

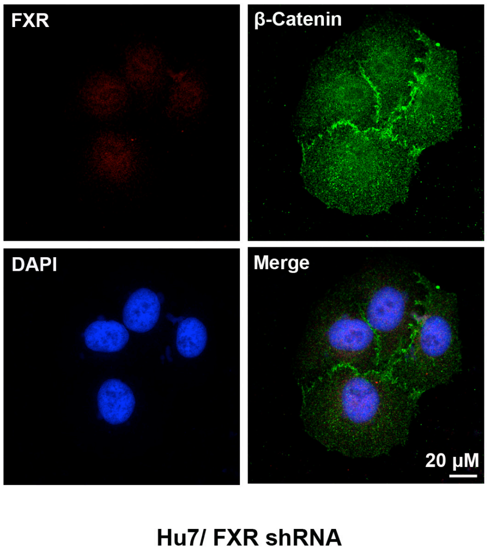

Supplementary Figure S2: β-Catenin distribution in Huh7/FXR shRNA cells. Huh7 cells stably expressing pLKO.1 shRNA FXR were double stained with monoclonal anti-FXR antibody and polyclonal β-Catenin antibody. Together with Figure 2D, knockdown of FXR expression did not change the localization of β-Catenin. Scale bar = 20 μm.

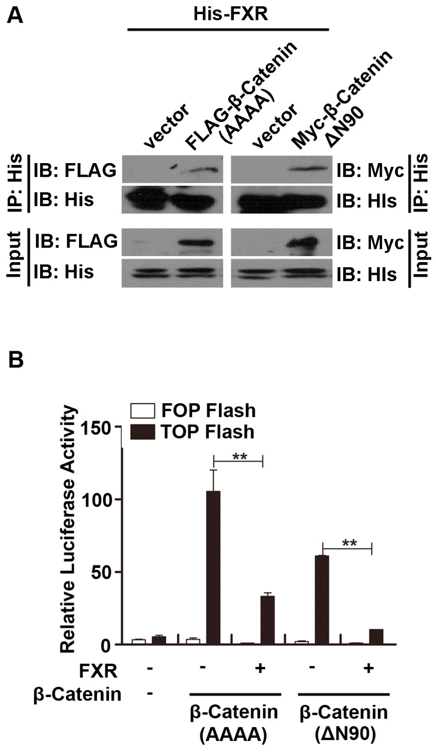

Supplementary Figure S3: FXR interacted with the oncogenic β-Catenin mutants and inhibited the transcriptional acitvity. (A) FXR bound with β-Catenin oncogenic mutants (S33A/S37A/T41A/S45A and ΔN90). Immunoprecipitation was carried out using Nickel beads in lysates from HEK293T cells co-transfected with His-FXR and FLAG-β-Catenin (S33A/S37A/T41A/S45A) or Myc-β-Catenin (ΔN90). β-Catenin mutants was detected using antibodies against FLAG or Myc respectively. **(B)** FXR suppressed β-Catenin oncogenic mutants (S33A/S37A/T41A/S45A and ΔN90) mediated transcriptional activity. HEK293T cells were transfected with TOPflash reporter (0.1 μg DNA), FXR and β-Catenin mutants (S33A/S37A/T41A/S45A or ΔN90). Luciferase activity was measured using cell lysates 24 hours after transfection. Reporter activity was normalized by pRL-TK luciferase activity. Error bars represent ± SEM of three independent samples. *, $p < 0.01$; **, $p < 0.001$.
